# Does trauma event type matter in the assessment of traumatic load?

**DOI:** 10.1080/20008198.2017.1344079

**Published:** 2017-07-06

**Authors:** Daniela Conrad, Sarah Wilker, Anett Pfeiffer, Birke Lingenfelder, Tracie Ebalu, Hartmut Lanzinger, Thomas Elbert, Iris-Tatjana Kolassa, Stephan Kolassa

**Affiliations:** ^a^ Clinical Psychology and Neuropsychology, Department of Psychology, University of Konstanz, Konstanz, Germany; ^b^ Clinical & Biological Psychology, Institute of Psychology and Education, Ulm University, Ulm, Germany; ^c^ VA Boston Healthcare System, Boston, MA, USA; ^d^ Institute of Number Theory and Probability Theory, Ulm University, Ulm, Germany; ^e^ SAP Switzerland AG, Tägerwilen, Switzerland

**Keywords:** Posttraumatic Stress Disorder, traumatic events, Random Forest Conditional Interference, Least Absolute Shrinkage and Selection Operator, PTSD risk, ranking, prediction

## Abstract

**Background**: The likelihood of developing Posttraumatic Stress Disorder (PTSD) depends on the interaction of individual risk factors and cumulative traumatic experiences. Hence, the identification of individual susceptibility factors warrants precise quantification of trauma exposure. Previous research indicated that some traumatic events may have more severe influences on mental health than others; thus, the assessment of traumatic load may be improved by weighting event list items rather than calculating the simple sum score.

**Objective**: We compared two statistical methods, Random Forests using Conditional Interference (RF-CI) and Least Absolute Shrinkage and Selection Operator (LASSO), based on their ability to rank traumatic experiences according to their importance for predicting lifetime PTSD.

**Methods**: Statistical models were initially fitted in a sample of *N*_1_ = 441 survivors of the Northern Ugandan rebel war. The ability to correctly predict lifetime PTSD was then tested in an independent sample of *N*_2_ = 211, and subsequently compared with predictions by the simple sum score of different traumatic event types experienced.

**Results**: Results indicate that RF-CI and LASSO allow for a ranking of traumatic events according to their predictive importance for lifetime PTSD. Moreover, RF-CI showed slightly better prediction accuracy than the simple sum score, followed by LASSO when comparing prediction results in the validation sample.

**Conclusion**: Given the expense in time and calculation effort by RF-CI and LASSO, and the relatively low increase in prediction accuracy by RF-CI, we recommend using the simple sum score to measure the environmental factor traumatic load, e.g., in analyses of gene × environment interactions.

## 1. Background

With increasing rates of conflict and terror, natural disasters, and modern wars, the number of humanitarian emergencies is rising and has reached highest numbers since World War II (United Nations High Commissioner for Refugees, 2015). Thus, a better understanding of the psychological consequences of traumatic events is of highest societal and scientific relevance. Many survivors of traumatic experiences develop trauma-spectrum disorders such as Posttraumatic Stress Disorder (PTSD), which is associated with severe individual suffering, impairments in daily functioning, elevated risk for diverse physical health impairments (Glaesmer, Brahler, Gündel, & Riedel-Heller, 2011; Kubzansky et al., 2014), and suicidality (Jakupcak et al., 2009).

Cumulative exposure to traumatic events has particularly grave consequences; as the number of traumatic events experienced (traumatic load) rises, the risk for PTSD increases in a ‘building-block’ manner (Schauer et al., 2003). Furthermore, PTSD prevalence rates reach up to 100% at extreme levels of trauma exposure (Kolassa, Ertl, Kolassa, Onyut, & Elbert, 2010; Neuner et al., 2004). However, only a significant minority of individuals develops PTSD at lower levels of traumatic load, indicating a high relevance of individual risk factors in predicting PTSD susceptibility. Important risk factors include demographic characteristics (e.g., Sayed, Iacoviello, & Charney, 2015), personality traits (e.g., Jakšić, Brajković, Ivezić, Topić, & Jakovljević, 2012; James et al., 2015), cognition and emotion regulation (e.g., Hayes, Vanelzakker, & Shin, 2012), genetic predispositions (e.g., DiGangi, Guffanti, McLaughlin, & Koenen, 2013; Wilker & Kolassa, 2013), and molecular mechanisms (e.g., Neumeister, Seidel, Ragen, & Pietrzak, 2015; Steudte-Schmiedgen et al., 2015; Van Zuiden, Kavelaars, Geuze, Olff, & Heijnen, 2013). These individual differences are similarly important for successful PTSD treatment (Bryant et al., 2008, 2010; Felmingham, Dobson-Stone, Schofield, Quirk, & Bryant, 2013; Wilker et al., 2014), and should therefore be considered in the allocation of therapeutic resources to individuals at high risk, as well as for the individualization of treatment.

However, due to the influence of traumatic load on PTSD risk, individual risk factors for PTSD development can be identified only if trauma exposure is simultaneously assessed. Therefore, the validity of the identified risk factors will strongly depend on the quality of the trauma assessment. Unfortunately, there are no clear indicators of how trauma exposure should be best quantified, e.g., in studies investigating gene × environment interactions. Wilker et al. (2015) previously raised the question whether the number of different traumatic event types is a reliable and valid predictor of lifetime PTSD, or whether event frequencies should be additionally considered to best measure PTSD risk. Since the more time-consuming assessment of event frequencies did not improve the accuracy of PTSD prediction, they recommended using the simple summation of the number of traumatic event types as a measurement for traumatic load.

Even though the simple sum score is assumed to serve as a useful proxy to measure traumatic load, some events may be more toxic than others. Netland (2005) suggested a weighting of event list items instead of an additive summation for traumatic load calculations to increase the accuracy of predictions on PTSD risk. Furthermore, ranking traumatic events according to their predictive importance for PTSD may allow for the exclusion of less predictive traumatic event types, and therefore save time and resources in diagnostic interviews. In a study conducted by Breslau,  Chilcoat,  Kessler,  and Davis (1999), the highest risks for PTSD were observed in individuals exposed to assaultive violence (e.g., military combat, rape, captivity, torture or kidnapping, being threatened by a weapon, being badly beaten). Additionally, the sudden unexpected death of a loved one was associated with a moderate risk for PTSD, while the experience of accidents, natural disasters, or witnessing others being killed or injured was associated with low conditional PTSD risk (Breslau et al., 1999). At least three other studies from different cultural settings have independently replicated these findings, showing that inter-personal, ‘man-made’ assaults more often lead to PTSD development than non-personal events (Ferry et al., 2014; Hapke, Schumann, Rumpf, John, & Meyer, 2006; Köbach, Schaal, & Elbert, 2015). In addition, Holbrook, Hoyt, Stein, and Sieber (2001) suggested that the perceived severity of a traumatic event may depend on the victim’s self-perceived fear of death.

However, previous investigations in various traumatized populations have used different event lists, making comparability between study populations difficult. Furthermore, empirical research explicitly testing the simple sum score against procedures accounting for the different pathogenicity of traumatic event is scarce. Köbach, Schaal, et al. (2015) recently employed the method of Random Forests embedded in a Conditional Interference framework (RF-CI) to prioritize event list items regarding their importance for predicting PTSD symptom severity. They investigated former male members of the Congolese armed groups and replicated their findings in a sample of Burundian ex-combatants (Köbach, Nandi et al., 2015). While both studies successfully identified certain events that were particularly important to PTSD symptom severity by RF-CI, best predictions were obtained using the simple sum score of all experienced events.

## 2. Objectives

The present study compared two different statistical procedures, RF-CI and the Least Absolute Shrinkage and Selection Operator (LASSO), in their ability to identify which traumatic events contribute most to PTSD development in a sample of Northern Ugandan rebel war survivors. Furthermore, predictions by the applied statistical models were compared to predictions using the simple sum score of traumatic events. Therefore, our study can provide practical advice for future trauma-related research studies applying event lists to assess multiple traumatic experiences. Knowing how to best measure trauma exposure may allow for a more precise assessment of other individual risk factors for PTSD. Additionally, in the long term, this may enable improvements in predicting treatment success, as well as the individualization and prioritization of treatments for patients with the highest therapeutic needs.

## 3. Method

### 3.1. Samples

Data was collected in the former Internal Displaced People (IDP) camps of Pabbo (Amuru District) and Koch Goma (Nwoya District) (*N* = 490), and in the re-settled communities and villages of the Gulu district (*N* = 240), Northern Uganda. Both areas were severely affected by the atrocities of the Lord’s Resistance Army (LRA), including abductions, forced recruitment, killings, mutilations, and sexual violence. All participants provided written informed consent and only participants who experienced at least one traumatic event were included in this study. Exclusion criteria from the data analyses were signs of current psychotic symptoms and reports of current alcohol abuse as this may have influenced the validity of interview responses. Furthermore, only individuals with non-missing data regarding PTSD diagnosis and traumatic events were considered. Both cohorts were independently sampled and will hereafter be referred to as the training and test sample.

#### 3.1.1. Training sample

For recruitment of the training sample, counsellors visited residents of the former IDP camps and communities at their homes, explaining the aim and scope of the research project. Interested individuals were invited for an interview. One participant in the training sample was significantly older (age = 80 years) than the others (age range 18–63 years); this participant was excluded from all analyses, because of a potential age-related memory bias for the experienced traumatic events. Furthermore, 48 participants of the training sample were removed due to missing data regarding lifetime PTSD diagnosis and events. The final training sample used to build the prediction models included 441 participants (254 female, *M*_age_ = 30.52, *SD*_age_ = 9.86, age range: 18–63 years).

#### 3.1.2. Test sample

For recruitment of the test sample, study procedures were introduced in community meetings. Community members interested in participating were invited for an interview. Of the 240 participants interviewed, 29 were excluded from the test sample: 10 showed signs of current alcohol abuse, one had a history of psychotic symptoms, two experienced difficulties in understanding interview questions, and 16 were excluded due to missing data on the event list. The test sample used to evaluate prediction accuracy consisted of 211 individuals (111 female, *M*_age_ = 33.60, *SD*_age_ = 10.67, age range: 18–62 years).

### 3.2. Materials and study procedure

All procedures followed the Declaration of Helsinki and were approved by the Institutional Review Board of Gulu University, Uganda, the Ugandan National Council for Science and Technology (UNCST), and the ethics committee of the German Psychological Society (Deutsche Gesellschaft für Psychologie, DGPs). Diagnostic interviews were performed by trained local counsellors who had received intensive training on the concepts of mental health disorders, trauma and PTSD, counselling skills, and quantitative data collection. Additionally, they were supervised by psychologists with specialization in psychotraumatology. All diagnostic instruments were translated into Luo, the local language of Northern Uganda, following a procedure of blind-translation and back-translation according to scientific standards. Trauma exposure was assessed using a 62-item event list, used for traumatic load assessment in previous studies (Wilker et al., 2014, 2015), and covered: (1) natural traumatic events (e.g., natural disasters), (2) events connected to war and violence in general (e.g., close to shelling or bomb attack), (3) LRA-specific events (e.g., forced to eat human flesh), (4) events where the victims were forced to become perpetrators themselves (e.g., forced to attack villages), (5) the experience of domestic violence (e.g., severely beaten by spouse), and (6) other traumatic events (e.g., accidents, life-threatening illness). For the diagnosis of lifetime PTSD according to DSM-IV-TR (American Psychiatric Association, 2000) the Posttraumatic Diagnostic Scale (PDS; Foa, Cashman, Jaycox, & Perry, 1997) was employed as a structured interview. The reliability and validity of the translated PDS applied by local interviewers has been previously documented (Ertl et al., 2010).

### 3.3. Statistical procedures

To investigate which traumatic events contribute the most to PTSD development, two different classification methods were performed: RF-CI and LASSO. Recent investigations by Kessler et al. (2014) indicated Random Forest as best machine learning approach to predict PTSD after trauma exposure. To evaluate prediction accuracy, the authors calculated the area under the receiver operator characteristics curve (AUC). Thus, AUC = 1 indicates perfect discrimination of cases from controls by the model, AUC = .5 presents a prediction accuracy not better than by chance, and AUC = 0 demonstrates the incorrect classification of all subjects (Hajian-Tilaki, 2013). In the study by Kessler et al. (2014), RF-CI comprised highest AUC with .96, while LASSO performed as good as other applied penalized regressions (e.g. Ridge and Elastic net) and logistic regression models (AUC = .90).

#### 3.3.1. Random Forests with Conditional Interference

Random Forests are a combination of multiple classification trees (Breiman, 2001). Each tree in the forest is built using a bootstrap sample. At each node, the tree splits on a small randomly chosen subset of the features (e.g., traumatic events) instead of a full feature set, choosing one feature to minimize an appropriate measure of impurity or misclassifications in the child nodes. This leads to faster model fitting compared to standard classification trees. Decisions in splitting nodes are binary and repeated until a terminal node is reached and determines the final response class (e.g., PTSD versus no PTSD). Given the multitude of random trees in a Random Forest, using random features as splitter variables, and bootstrapped samples to build each tree, the potential of over-fitting a single tree is minimized. Furthermore, the correlation between trees is reduced, without increasing the variance too much). Thus, in contrast to standard classification trees, the trees in a Random Forest are not pruned. In addition, Random Forests allow for predictions on independent test data, by feeding a new feature vector into each of the forest’s component trees and having the component trees vote on the final classification. Furthermore, information about the importance of a variable is provided. For this, each tree is applied to those cases that were *not* chosen in this tree’s bootstrap sample (so-called out-of-bag [OOB] data), but with one feature randomly permuted among cases. The deterioration in classification accuracy between the original and the permuted data is reported as this feature’s importance: if permuting a feature’s values does not reduce classification accuracy much, then it is *ipso facto* unimportant for classification (Breiman, 2001). However, the original permutation importance measure in Random Forests overestimates the importance of predictor variables that are correlated. Therefore, Strobl, Boulesteix, Kneib, Augustin, and Zeileis (2008) suggested the calculation of conditional variable importance for Random Forests (RF-CI). RF-CI reflects the true impact of each predictor more reliably than the original marginal approach and similarly allows for the consideration of potential confounding factors, by performing the permutation in importance assessment only conditional on the values of other features that the feature under investigation is correlated with.

#### 3.3.2. Least Absolute Shrinkage and Selection Operator

LASSO describes another popular model selection and shrinkage estimation method, originally proposed for linear regression models (Tibshirani, 1996), but similarly applicable to the logistic case (Genkin, Lewis, & Madigan, 2007; Hastie, Tibshirani, & Wainwright, 2015; Roth, 2004). LASSO was introduced as the estimation of regression coefficients using maximum likelihood; however, this can lead to data overfitting, resulting in poor predictive accuracy due to high variance in the predicted values (Genkin et al., 2007). Furthermore, interpretation of results can be difficult as all independent variables (e.g., all events in the event list) will be included in the regression model (*β_j_* ≠ 0).

LASSO includes an additional L^1^-norm penalty parameterized by a weight 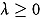, which is usually set by cross-validation, and minimizes the sum of the negative log-likelihood and the penalty term. A LASSO model will set some of the regression coefficients exactly to zero, depending on the value chosen for 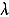 (Tibshirani, 1996). Note that this is a non-trivial result, which depends crucially on the use of an L^1^ norm: the analogous regularization using an L^2^ norm, or *ridge regression*, will not have this property. Thus, a LASSO model will only involve a subset of predictors with non-zero coefficients, leading to more interpretable models with improved prediction accuracy for independent samples (Tibshirani, 1996). Furthermore, statements about the importance of variables can be made: the earlier a variable is taken into the regression model as 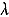 reduces from very large positive values to zero, the more important the variable is as predictor.

### 3.4. Data analysis

All statistical analyses were performed in the statistical environment RStudio version 3.2.1 (RStudio Team, 2015), applying the R package ‘party’ version 1.0.25 for RF-CI (Hothorn, Buhlmann, Dudoit, Molinaro, & van der Laan, 2006; Strobl et al., 2008; Strobl, Boulesteix, Zeileis, & Hothorn, 2007), and ‘glmnet’ version 2.0.2 for LASSO computations (Friedman, Hastie, & Tibshirani, 2010).

Calculating conditional importance using all 62 events as predictors resulted in a bug, which is – according to the package developers – most likely due to the large number of categorical predictors included, causing a too high complexity of the conditioning grid. They suggested to calculate the unconditional importance instead, previously indicated to resemble the behaviour of the conditional importance, with increasing number of randomly preselected splitter variables per node (mtry; Strobl et al., 2008). In order to determine the optimal number of splitter variables, Random Forests were built on the training data with 500 trees each. Simulations were repeated 101 times using different random number generator seeds for different values of the mtry parameter. This number ranged between the square of the total number of predictors (= 8; default value for Random Forest classifications; Hastie, Tibshirani, & Friedman, 2009), and one-third of the total number of predictors (= 21; default value for Random Forest regressions; Hastie et al., 2009). Comparing the averaged simulation results of all 14 values tested, the best model fit regarding mean prediction accuracy and standard deviation in the full training sample was provided by mtry = 20, henceforth used for the analyses in the test sample. To similarly overcome the problem of correlated predictors in LASSO regression, cross-validation was applied as recommended by Hebiri and Lederer (2012). Possibly due to unbalanced numbers of PTSD cases and controls in the samples, cross-validated mean squared errors resulted in more economical models, with less predictors and better prediction accuracy than the cross-validation of the misclassification error. Assuming that some events could be experienced differently based on gender, both models were additionally calculated for males and females separately. For better comparability with results proposed by Köbach, Nandi et al. (2015) and Köbach, Schaal et al. (2015), distinct calculations were further performed for the group of ex-combatants and non-combatants. Groups were classified by the event list item: *Have you ever been fighting in combat* (yes/no). The group of former combatants comprised 118 (26.76%) individuals in the training sample and 51 (24.17%) individuals in the test sample, while the group of non-combatants consisted of 323 (73.24%) individuals in the training sample and 160 (75.83%) in the test sample.

To evaluate prediction accuracy, predictions of lifetime PTSD diagnosis for an independent sample were separately obtained by all models built with RF-CI and LASSO, respectively. For RF-CI, the voting majority of all 101 models were compared with the actual diagnosis, while for LASSO, the mean of the predicted probabilities was computed, classifying values higher than or equal to 0.5 as PTSD, and values below 0.5 as no PTSD. This procedure was similarly applied for traumatic load, which was calculated as the number of different traumatic event types experienced and entered in a logistic regression. Evaluation parameters for the predictions were: accuracy (correctly diagnosed cases and controls divided by total sample size), sensitivity (proportion of correctly identified PTSD cases), and specificity (proportion of correctly identified controls).

## 4. Results

A total of 78% (*n* = 346) of the participants in the training sample and 72% (*n* = 151) in the test sample fulfilled the diagnostic criteria for lifetime PTSD. Only 24% (*n* = 104) in the training sample and 22% (*n* = 47) in the test sample met the criteria for a current PTSD diagnosis. All participants had experienced multiple traumatization with an average of 26.45 events (*SD* = 8.41, range: 9–55) in the training sample and 26.87 events (*SD* = 9.38, range: 2–61) in the test sample. Frequencies by which traumatic events were experienced in each sample can be found in the Supplemental data (Supplementary Table 2 and Table 3).

**Figure 1.**
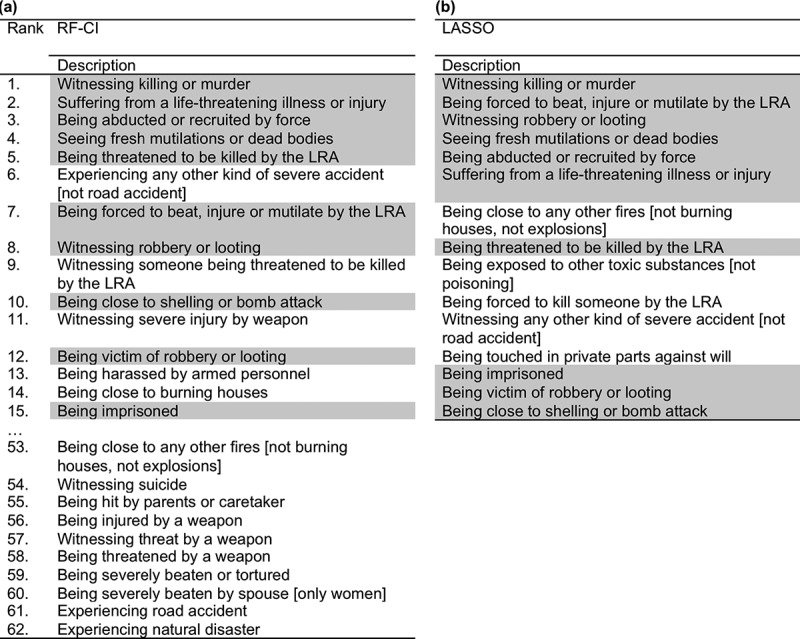
Ranking of traumatic events in decreasing order according to (a) RF-CI and (b) LASSO applied on the training sample (*N* = 441). Events within the first 15 ranks similarly found by RF-CI and LASSO are highlighted in grey. Note: Only the first 15 and last 10 ranks of RF-CI are displayed. RF-CI = Random Forest Conditional Interference; LASSO = Least Absolute Shrinkage and Selection Operator.

RF-CI and LASSO were performed to investigate which specific events are the most important predictors for PTSD development. Figure 1 displays the ranking of the events in decreasing order, separately listed for RF-CI and LASSO. As LASSO reduces the number of predictors by setting some coefficients exactly to zero, the results comprised a subset of 15 predictors, while RF-CI results contained all 62 events. Within the first 15 ranks of RF-CI and LASSO, ten matches were found, including six events related to war and violence in general (*Witnessing killing or murder, Witnessing robbery or looting, Seeing fresh mutilations or dead bodies, Being abducted or recruited by force, Being imprisoned, Being close to shelling or bomb attack*), two LRA-specific events (*Being threatened to be killed by the LRA, Being forced to beat, injure or mutilate someone by the LRA*), and two events independent of war and violence (*Suffering from a life-threatening illness or injury, Witnessing any other kind of severe accident [not road accident]*). Even though the order of events slightly differed between RF-CI and LASSO, both models identically indicated *Witnessing killing or murder* to be the most important predictor for PTSD.

Prediction results are displayed in Table 1 and were evaluated with regard to overall prediction accuracy, sensitivity, and specificity. Best overall prediction accuracy was provided by RF-CI, followed by traumatic load, and LASSO, respectively. In the logistic regression using traumatic load, PTSD diagnoses were assigned to participants with 16 or more different traumatic event types experienced. Predictions of all models were superior to the intercept-only model.

**Table 1.** Correct predictions (in %) for RF-CI, LASSO and traumatic load.

	RF-CI	Traumatic load	LASSO
Accuracy	77.25	75.36	74.88
Error rate	22.75	24.64	25.12
Sensitivity	98.01	94.04	94.04
Specificity	25.00	28.33	26.67

Predictions on the independent test sample (*N* = 211). RF-CI = Random Forest Conditional Interference; LASSO = Least Absolute Shrinkage and Selection Operator.

Assuming that some events could be experienced differently based on gender, RF-CI and LASSO were subsequently calculated separately for males and females. Data analysis showed that the ranking order of events differed between males and females. While for females *Witnessing killing or murder* consistently remained the most important predictor, *Seeing fresh mutilations or dead bodies* (RF-CI) and *Being threatened to be killed by the LRA* (LASSO), appeared to be the strongest predictors for PTSD development for males. Again, RF-CI revealed better overall prediction accuracy (males = 73.00%, females = 76.58%) than LASSO (males = 71.00%, females = 73.87%). However, compared with the overall sample, prediction accuracy did not improve with gender-specific RF-CI models. While prediction sensitivity could be increased, models showed strong decreases in prediction specificity for both males (sensitivity = 98.55%, specificity = 16.13%) and females (sensitivity = 100.00%, specificity = 10.35%). Separate calculations for the group of individuals who were former combatants, resulted in a ranking order of events completely distinct from those who were non-combatants. The three most important predictors for lifetime PTSD development in former combatants were *Being threatened by a weapon, Being raped*, and *Being forced to abduct children or adults*, while *Witnessing killing or murder, Suffering from a life-threatening illness,* and *Seeing fresh mutilations or dead bodies* appeared to be the worst for non-combatants. While with a separate model for former combatants, the prediction accuracy increased to 86.27%, none of the healthy controls was correctly predicted (sensitivity = 100.00%, specificity = 0%). However, no increase in prediction accuracy was observed for the group of non-combatants (accuracy = 73.13%, sensitivity = 96.26%, specificity = 26.42%).

## 5. Discussion

The aim of this study was to investigate how trauma exposure could be best quantified and henceforth included in trauma-related research, in order to allow for a precise assessment of other individual risk factors – such as genetic factors – for lifetime PTSD development. We used two different statistical models, RF-CI and LASSO, to rank traumatic event types according to their predictive importance for lifetime PTSD risk in Northern Ugandan rebel war survivors. Then, we compared the prediction results of both models with predictions of the simple sum score of traumatic events experienced. The event list utilized included 62 traumatic events, containing inter-personal trauma related to war in general and the LRA-war in specific, war-independent violence, as well as domestic violence, and natural trauma. The ranking by RF-CI included all 62 events, whereas LASSO consisted only of a subset of predictors. Within the first 15 ranks of RF-CI and LASSO, 10 events were found for both models. Of those 10 events, eight were related to war and violence in general and specifically to the LRA war. Therefore, our results replicate previous findings demonstrating that the experience of war-related events, specifically the (threatening) death or injury of others or self, increases the risk for PTSD (Holbrook et al., 2001). On the contrary, only two events described non-man-made trauma (*Suffering from a life-threatening illness or injury* and *Witnessing any other kind of severe accident [not road accident]*).

Both RF-CI and LASSO identified *Witnessing killing or murder* to be the most important predictor for the development of PTSD in the investigated study population. However, some of the events frequently indicated by the participants to be the worst experienced (see Supplementary Table 2 and Table 3), and thus presumed to contribute significantly to PTSD development, did not appear within the first ranks of RF-CI (e.g., *Being forced to kill someone by the LRA*). Ferry et al. (2014) suggested that although some events highly increase the conditional PTSD risk, their contribution to the overall burden of PTSD might be rather small, as they show comparatively low prevalence in the overall population. Indeed, the number of people who were *Being forced to kill someone by the LRA* amounted to only 97 in the training sample (22.00%) and 33 (15.64%) in the test sample. However, among those who experienced this event, 66 individuals in the training sample (68.04%) and 23 in the test sample (69.70%) reported this to be the worst event ever experienced. Furthermore, events that participants subjectively identify as the worst do not necessarily have the highest PTSD risk. If the events are accompanied by feelings of guilt, they may be experienced more severely by the individual than events with objectively higher life threat. For example, RF-CI revealed a completely different list of the most important events for former combatants than non-combatants.

Regarding prediction accuracy for lifetime PTSD in an independent sample, the best results were found using RF-CI, followed by traumatic load and LASSO, respectively. Thus, our results are in contradictions with previous findings on PTSD symptom severity in OOB data by Köbach, Nandi et al. (2015) and Köbach, Schaal et al. (2015). The authors compared RF-CI and traumatic load in two independent African populations of male ex-combatants and found better prediction results by the simple sum score of traumatic event types experienced than by RF-CI. Since our samples included males and females, in contrast to Köbach, Schaal et al. (2015) and Köbach, Nandi et al. (2015), analyses were computed separately for both genders and the groups of combatants and non-combatants. However, prediction accuracy did not increase by fitting gender-specific or group-specific models.

To summarize, this study demonstrated that RF-CI results were superior to the prediction accuracy obtained by the simple sum score and LASSO. Thus, our results indicate that weighting event list items according to RF-CI, before calculating the traumatic load, could improve the quantification of traumatic experiences in trauma-related research and their differentiation from other individual risk factors. However, the increase in prediction accuracy by RF-CI only amounted to 1.89%. Given the expense in time and computational effort, a simple summation of the traumatic event types experienced may be more economical. However, the reliable and valid assessment of traumatic load requires the identification of all events an individual may have experienced. Given the fact that a full bundle of questionnaires including a multi-page event list might not be applicable in a diverse study setting, their shortening may be worthwhile. Thus, LASSO could represent a possible approach to not only rank event list items, but also reduce them according to their importance for PTSD risk. However, in the present study, prediction accuracy of LASSO was slightly lower than by traumatic load and RF-CI.

Even though our results have relevant implications for future research, some limitations must be addressed. While the number of healthy individuals in the test sample amounted to only 28% compared with 72% diagnosed with lifetime PTSD, the prediction specificity of all procedures applied was rather small. Future studies should therefore aim for more out-balanced samples. Furthermore, all participants in this study were survivors of the LRA war in Northern Uganda, and the event list used was specifically designed for this context. Thus, our results may not be generalizable to other study cohorts. It is indeed very likely that the ranking of traumatic events will vary with other investigated populations as being dependent on the traumatic events asked for in each study. To cover this problem, future studies may use latent class analyses (LCA). Different than variable-centred approaches (e.g., RF-CI and LASSO), LCA is a person-centred approach that forms mutually exclusive classes of individuals with similar response patterns. However, the primary goal of this study was to provide practical advice on how to improve the assessment of traumatic load and subsequent PTSD predictions by suited statistical measures such as RF-CI and LASSO, that are applicable to various traumatized populations. Which procedure is ‘best’ suited to assess traumatic load and other risk factors for PTSD development depends on the researchers’ and clinicians’ aims, which may be a more accurate, more economic or more time-efficient assessment of traumatic load. Independent of the approach used, one should not underestimate that for an individual some events may be of high importance, even though they do not appear within the first ranks of the statistical procedures, such as RF-CI and LASSO.

## Highlights of the article

- We investigated whether ranking of traumatic events may improve traumatic load assessment rather than using the simple sum score of experienced event types.
- Both statistical models compared (RF-CI and LASSO) allow for a ranking of traumatic events and revealed similar results regarding their predictive importance for PTSD development.
- Prediction accuracy of PTSD risk was only slightly improved when ranking events by RF-CI and is accompanied with expenses in time and calculation effort.

## Supplementary Material

Supplementary material and Chinese/Spanish abstracts
