# Supplementary material for: Does trauma event type matter in the assessment of traumatic load?
Source: Eur J Psychotraumatol. 2017 Jul 6;8(1):1344079. doi: 10.1080/20008198.2017.1344079 (PMC5533143; doi:10.1080/20008198.2017.1344079)
Supplement: Supplementary material and Chinese/Spanish abstracts [file zept_a_1344079_sm3211.zip › Supplements.docx]

# Supplement

Supplementary Table 1*.*

*Frequencies of events, PTSD cases, and worst event ratings related to overall frequency in decreasing order (training sample).*

| Rank Description | | Overall frequency (%) | | PTSD cases | Rating “worst event” (%) | |
| --- | --- | --- | --- | --- | --- | --- |
| 1. | Being forced to kill someone by the LRA | 97 | (22.00) | 96 | 66 | (68.04) |
| 2. | Witnessing killing or murder | 290 | (65.76) | 257 | 119 | (41.04) |
| 3. | Experiencing a traumatic event not included in list* | 33 | (7.48) | 31 | 6 | (18.18) |
| 4. | Seeing people with fresh mutilations or dead bodies | 375 | (85.03) | 308 | 67 | (17.87) |
| 5. | Being severely beaten or tortured | 292 | (66.21) | 239 | 25 | (8.56) |
| 6. | Being forced to skin, chop or cook dead bodies by the LRA | 29 | (6.58) | 29 | 2 | (6.90) |
| 7. | Being threatened to be killed by the LRA | 333 | (75.51) | 280 | 21 | (6.31) |
| 8. | Being injured by a weapon | 164 | (37.19) | 139 | 10 | (6.10) |
| 9. | Being raped | 82 | (18.59) | 73 | 5 | (6.10) |
| 10. | Being close to crossfire or shooting of snipers | 358 | (81.18) | 289 | 19 | (5.31) |
| 11. | Fighting in combat | 118 | (26.76) | 111 | 5 | (4.24) |
| 12. | Being bewitched | 119 | (26.98) | 101 | 5 | (4.20) |
| 13. | Being forced to eat human flesh by the LRA | 25 | (5.67) | 25 | 1 | (4.00) |
| 14. | Being close to a combat situation | 429 | (97.28) | 337 | 14 | (3.26) |
| 15. | Being close to shelling or bomb attack | 381 | (86.39) | 310 | 12 | (3.15) |

*Note.* The table displays only the first 15 ranks of events, sorted in decreasing order by the relative frequency of being rated as the worst event experienced by the individuals in the training sample. The ratings of five participants were excluded as they indicated two worst events instead of one. *Events in this category could not be assigned to other items in the list due to divergent content-related details.

Supplementary Table 2.

*Frequencies of events, PTSD cases, and worst event ratings related on overall frequency in decreasing order (test sample).*

| Rank Description | | Overall frequency (%) | | PTSD cases | Rating “worst event” (%) | |
| --- | --- | --- | --- | --- | --- | --- |
| 1. | Being forced to kill someone by the LRA | 33 | (15.64) | 31 | 23 | (69.70) |
| 2. | Being abducted or recruited by force | 129 | (61.14) | 104 | 55 | (42.64) |
| 3. | Experiencing a traumatic event not included in list* | 21 | (9.95) | 15 | 8 | (38.10) |
| 4. | Seeing people with fresh mutilations or dead bodies | 190 | (90.05) | 141 | 42 | (22.11) |
| 5. | Fighting in combat | 51 | (24.17) | 44 | 5 | (9.80) |
| 6. | Being severely beaten or tortured | 131 | (62.09) | 103 | 9 | (6.87) |
| 7. | Being forced to beat, injure, mutilate someone by the LRA | 59 | (27.96) | 53 | 4 | (6.78) |
| 8. | Being threatened by a weapon | 122 | (57.82) | 99 | 8 | (6.56) |
| 9. | Being burned by parents or caretakers on purpose | 16 | (7.58) | 11 | 1 | (6.25) |
| 10. | Being raped | 34 | (16.11) | 32 | 2 | (5.88) |
| 11. | Being forced to skin, chop or cook dead bodies by the LRA | 18 | (8.53) | 18 | 1 | (5.56) |
| 12. | Being injured by a weapon | 69 | (32.70) | 57 | 3 | (4.35) |
| 13. | Being harassed by armed personnel | 139 | (65.88) | 109 | 5 | (3.60) |
| 14. | Being severely beaten by spouse (only women)^1^ | 60 | (28.44) | 44 | 2 | (3.33) |
| 15. | Being close to a combat situation | 192 | (91.00) | 143 | 6 | (3.13) |

*Note.* The table displays only the first 15 ranks of events, sorted in decreasing order by the relative frequency of being rated as the worst event experienced by the individuals in the test sample. One participant indicated an event to be the worst experienced that was not included in the event list, and thus was excluded. ^1^Females in population *n*=111. *Events in this category could not be assigned to other items in the list due to divergent content-related details.
