# Supplementary material for: Does trauma event type matter in the assessment of traumatic load?
Source: Eur J Psychotraumatol. 2017 Jul 6;8(1):1344079. doi: 10.1080/20008198.2017.1344079 (PMC5533143; doi:10.1080/20008198.2017.1344079)
Supplement: Supplementary material and Chinese/Spanish abstracts [file zept_a_1344079_sm3211.zip › Chinese abstract.pdf]

Article Title: Does trauma event type matter in the assessment of traumatic load?

Iris-Tatjana Kolassa; Daniela Conrad; Sarah Wilker; Anett Pfeiffer; Birke Lingenfelder; Tracie Ebalu; Hartmut Lanzinger; Thomas Elbert; Stephan Kolassa

题目：创伤事件类型对评估创伤负担是否重要？

背景：发生创伤后应激障碍（PTSD）的可能性取决于个体的风险因子和创伤经验累积的交互作用。所以对个体易感性的识别可以保证对创伤暴露的精确量化。前人研究表明有的创伤事件和其它类型相比，可能对精神健康有更严重的影响。因此，对事件清单项目进行加权计算而不是简单进行加总，可能会改进对创伤负担的评估。

目的：我们使用了两种统计方法：随机森林使用条件干扰（Random Forests using conditional interference，RF-CI) 和最小绝对值收敛和选择算子（Least Absolute Shrinkage and Selection Operator，LASSO），将创伤经验根据其预测终生PTSD的重要性进行排序，并根据这种排序能力对两种方法进行比较。

方法：首先将统计模型拟合于一个北乌干达叛乱战争的幸存者样本数据（N1 = 441），然后在一个独立的样本（N2 = 211）上进行验证，再将其与对不同创伤事件类型进行简单加总的方法并对终生PTSD的准确预测能力进行比较。

结果：结果表明RF-CI和LASSO可以根据预测终生PTSD的重要性对创伤经验进行排序。在验证样本中比较预测结果时RF-CI优于LASSO，并显示了比简单加总法略微较好的预测准确性。

结论：考虑到使用RF-CI和LASSO的时间投入和计算量，加上RF-CI只能较小地提高预测准确度，我们建议使用简单加总的方法来测量环境因子的创伤负担（比如在分析基因× 环境交互作用时）。

关键词：创伤后应激障碍，创伤事件，随机森林条件干扰，最小绝对值收敛和选择算子，PTSD风险，排序，预测
