# Supplementary material for: Does trauma event type matter in the assessment of traumatic load?
Source: Eur J Psychotraumatol. 2017 Jul 6;8(1):1344079. doi: 10.1080/20008198.2017.1344079 (PMC5533143; doi:10.1080/20008198.2017.1344079)
Supplement: Supplementary material and Chinese/Spanish abstracts [file zept_a_1344079_sm3211.zip › Spanish abstract.pdf]

¿Importa el tipo de acontecimiento traumático en la evaluación de la carga traumática?

Antecedentes: La posibilidad de desarrollar un trastorno por estrés postraumático (TEPT) depende de la interacción entre factores de riesgo individuales y experiencias traumáticas acumulativas. Por tanto, la identificación de los factores de susceptibilidad individual garantiza una cuantificación precisa de la exposición al trauma. Los estudios previos indican que algunos acontecimientos traumáticos pueden tener una influencia más grave que otros sobre la salud mental. Por tanto, la evaluación de la carga traumática puede mejorarse dándole peso a los ítems de la lista de acontecimientos en lugar de calcular una suma simple de las puntuaciones.

Objetivo: Comparamos dos métodos estadísticos, los bosques aleatorizados usando interferencia condicional (RF-CI, por sus siglas en inglés) y el método LASSO *(Least Absolute Shrinkage and Selection Operator),* por su capacidad para puntuar experiencias traumáticas en función de su importancia para predecir un TEPT de por vida.

Métodos: Inicialmente se probaron los modelos estadísticos en una muestra de N1= 441 supervivientes de la guerra rebelde de Uganda del Norte. Luego se comprobó la capacidad de predecir correctamente el TEPT crónico en una muestra independiente de N2 = 211, y se comparó posteriormente con las predicciones de la suma simple de puntuaciones de los diferentes tipos de acontecimientos traumáticos experimentados.

Resultados: Los resultados indican que RF-CI y LASSO permiten una clasificación de acontecimientos traumáticos de acuerdo a su importancia predictiva para el TEPT de por vida. Por otra parte, RF-CI mostró una precisión de predicción ligeramente mejor que la suma simple de las puntuaciones, seguido de LASSO al comparar los resultados de predicción en la muestra de validación.
Conclusión: Dado el gasto en tiempo y esfuerzo de cálculo que conllevan RF-CI y LASSO, y el relativamente bajo aumento en la precisión de la predicción por RF-CI, se recomienda el uso de la suma simple para medir la carga traumática del factor ambiental, por ejemplo, en los análisis de las interacciones gen x medioambiente.
